# Curcumin upregulates transforming growth factor-β1, its receptors, and vascular endothelial growth factor expressions in an in vitro human gingival fibroblast wound healing model

**DOI:** 10.1186/s12903-021-01890-9

**Published:** 2021-10-17

**Authors:** Auspreeya Rujirachotiwat, Supaporn Suttamanatwong

**Affiliations:** 1 Graduate Program in Pediatric Dentistry, Faculty of Dentistry, Chulalongkorn University, Bangkok, 10330 Thailand; 2 Present Address: Banphue Hospital, 134 Moo 2, Plubphue Road, Banphue District, Udonthani, 41160 Thailand; 3 Department of Physiology, Faculty of Dentistry, Chulalongkorn University, Bangkok, 10330 Thailand

**Keywords:** Curcumin, Gingiva, Fibroblasts, Wound healing, Vascular endothelial growth factor, Transforming growth factor beta

## Abstract

**Background:**

Curcumin accelerates healing of oral wounds; however, the responsible mechanisms remain underexplored. Our hypothesis is curcumin regulates the expression of wound healing-related genes in human gingival fibroblasts (hGFs). This study investigated whether curcumin regulates transforming growth factor (TGF)-β1, type I TGF-β receptor (TGF-βRI), type II TGF-β receptor (TGF-βRII), and vascular endothelial growth factor (VEGF) expression in unwounded hGFs and an in vitro hGF wound healing model.

**Methods:**

The cytotoxicity of curcumin was evaluated using the MTT assay. Unwounded hGFs were treated with non-cytotoxic concentrations of curcumin for 24 h. Gene expression was determined by quantitative polymerase chain reaction. Then, hGFs were treated with 1 µM curcumin in an in vitro wound healing model. PD98059 pretreatment was performed to determine whether extracellular signal-regulated kinase (ERK) signaling was required for regulation of gene expression by curcumin.

**Results:**

Curcumin at 0.1–20 µM caused no significant change in cell viability. In unwounded hGFs, curcumin had no significant effect on TGF-β1, TGF-βRI, TGF-βRII, or VEGF expression. Conversely, curcumin significantly upregulated the expression of these genes in the in vitro wound healing model. PD98059 significantly attenuated the curcumin-stimulated TGF-βRI, TGF-βRII, and VEGF expression, whereas it had no effect on TGF-β1 expression.

**Conclusions:**

Curcumin upregulated TGF-β1, TGF-βRI, TGF-βRII, and VEGF expression in an in vitro hGF wound healing model. The ERK pathway is required for TGF-βRI, TGF-βRII, and VEGF induction by curcumin. Our findings support the development of curcumin as a therapeutic agent for gingival ulcers.

## Background

Gingival wound healing is a complex process regulated by signals from several cell types, including immune cells, fibroblasts, endothelial cells, and keratinocytes [1]. The wound healing processes comprises four stages: (1) hemostasis, (2) inflammation, (3) proliferation, and (4) remodeling [2]. Gingival fibroblasts play an important role in the proliferative phase of wound healing by secreting multiple cytokines, growth factors, and extracellular matrix, including transforming growth factor beta (TGF-β), vascular endothelial growth factor (VEGF), epidermal growth factor, fibroblast growth factor, and collagen [3], all of which are critical for wound healing [4].

TGF-β, the prototype cytokine of its family, is produced by activated macrophages, platelets, keratinocytes and fibroblasts [5–8]. The cytokine plays important roles in regulating many cellular functions [9]. Specifically, TGF-β1 promotes wound healing by initiating inflammation, forming granulation tissue, and stimulating collagen synthesis and wound contraction [10–12]. Although the three TGF-β isoforms (TGF-β1, TGF-β2, and TGF-β3) share 60–80% structural similarity, they are encoded by different genes. These isoforms are secreted as inactive molecules that are activated prior to binding to their specific receptors, type I TGF-β receptor (TGF-βRI) and type II TGF-β receptor (TGF-βRII) [13].

The VEGF family comprises VEGF-A, VEGF-B, VEGF-C, VEGF-D, and placental growth factor [14]. VEGF is produced by platelets, neutrophils, endothelial cells, fibroblasts, and macrophages [15–19]. VEGF was initially identified as a vascular permeability factor that recruits inflammatory cells. VEGF is upregulated during wound healing [20]. VEGF promotes angiogenesis and granulation tissue formation in the proliferative phase of wound healing by stimulating endothelial cells to proliferate and form new blood vessels [21]. VEGF also has critical roles in inflammation, re-epithelialization, and scar tissue formation [21].

Curcumin (diferuloylmethane), which belongs to the curcuminoid family, is a major constituent of turmeric rhizome that is responsible for its yellow color [22]. Curcumin has long been used as a spice and medicinal herb [23]. Curcumin has anti-bacterial, anti-inflammatory, anti-oxidant, and anti-carcinogenic properties [24–27]. Curcumin has been shown to stimulate dermal and oral wound healing in animal models and clinical studies [28–32]. Curcumin promoted fibroblast proliferation and increased the level of antioxidant enzymes in rat dermal wounds [28, 31]. Furthermore, curcumin enhanced collagen production and reduced matrix metalloproteinase-9 production in rat cutaneous wounds [32]. When used to treat oral wounds, curcumin accelerated the healing of mucosal ulcers on the upper labial gingiva of rabbits [29]. Clinically, topical curcumin gel notably reduced the size of minor aphthous ulcers compared with a placebo [30]. Although curcumin has demonstrated remarkable wound healing properties, the cellular response to curcumin treatment during oral wound healing remains unclear.

Previous studies found that curcumin regulated gene expression by modulating enzyme activity and signaling pathways, such as mitogen-activated protein kinase (MAPK) [33, 34]. However, little is known about the effects of curcumin on gene expression in gingival fibroblasts and the responsible signaling pathways. Therefore, we examined the effect of curcumin and extracellular signal-regulated kinase (ERK) inhibitor on TGF-β1, TGF-βRI, TGF-βRII, and VEGF expression using an in vitro human gingival fibroblast (hGF) wound healing model.

## Methods

### Cell culture

HGFs were isolated from the healthy gingival tissue explants of three donors (two men and one woman; mean age, 21.3 years) who underwent surgical extraction of their third molars. Informed consent forms were obtained from all subjects. The study protocol was approved by the Ethics Committee of the Faculty of Dentistry, Chulalongkorn University. 

Isolation of hGFs was performed as previously described [35]. Briefly, the explants were immediately transferred in ice-cold Dulbecco’s Modified Eagle’s Medium (DMEM) (Sigma-Aldrich, St. Louis, MO, USA) containing 10% fetal bovine serum (FBS, Gibco, Waltham, MA, USA). The collected gingival tissues were washed with phosphate-buffered saline (PBS, Gibco). The specimens were cut into 1 mm^2^ pieces and cultured in DMEM containing 10% FBS at 37 °C in a humidified 5% CO_2_ atmosphere. Primary hGFs from the third to fifth passage were used in the experiments. Three donor cell lines were used in each independent experiment.

### Curcumin preparation

Curcumin (Sigma-Aldrich) was dissolved in dimethyl sulfoxide (DMSO, Sigma-Aldrich), due to its very low solubility in water, per the manufacturer’s instructions. The final concentration of DMSO was 0.1% in all experiments.

### Cell viability assay

HGFs were seeded at 5 × 10^3^ cells/well in 96-well flat-bottomed tissue culture plates in DMEM containing 10% FBS for 24 h. The medium was replaced with serum-free DMEM containing 0–50 µM curcumin (Sigma-Aldrich), and cells were incubated for another 24 h. Cell viability was determined using the MTT assay. Briefly, the medium was removed, 100 µL of 0.7 mg/mL MTT (Invitrogen, Waltham, MA, USA) solution in serum-free DMEM was added into each well, and the plates were incubated for 90 min until formazan crystal formation was microscopically visible. At the end of the incubation period, the MTT solution was removed, and 100 µL of dimethyl sulfoxide (DMSO, Sigma-Aldrich) was added to the wells and gently mixed to solubilize the formazan crystals. The absorbance of the dye was measured using a plate reader (EZ Read 400; Biochrom Cambridge, UK) at 570 nm. Cell viability was calculated using the following formula:

$${\text{Cell viability (\% )}} = ({\text{mean experimental absorbance/mean control absorbance}}) \times {1}00$$

### Gene expression and qPCR analysis

Gene expression was determined as previously described [35]. Briefly, hGFs were seeded at 6 × 10^5^ cells per 60-mm dish in DMEM containing 10% FBS. The following day, the cells were washed with PBS, and the medium was replaced with serum-free DMEM for 24 h. The cells were then incubated with 0–20 µM curcumin for 24 h. Then, total RNA extraction was performed and gene expression was evaluated by quantitative polymerase chain reaction (qPCR) as previously described [35]. Briefly, total RNA was extracted and 2 µg of total RNA from each sample was reverse transcribed. The cDNA template was amplified for 45 cycles. The primers for qPCR are listed in Table 1. Each reaction was conducted in duplicate. The specificity of the PCR products was verified by agarose gel electrophoresis and melting curve analysis. The expression of each gene was normalized to GAPDH expression using the 2^−ΔΔCt^ method. The gene expression shown in each figure represents the average of the values from three independent experiments. The qPCR and gene expression analysis were initially performed for all curcumin doses, then the most effective concentration was selected for analysis of the wound healing model.

**Table 1:** Primer sequences used for qPCR

Gene	Primer sequence
TGF-β1	Forward: 5′-GGATACCAACTATTGCTTCAGCTCC-3′ Reverse:5′-AGGCTCCAAATGTAGGGGCAGGGCC-3′
TGF-βRI	Forward: 5′-GGTCTTGCCCATCTTCACAT-3′ Reverse: 5′-TCTGTGGCTGAATCATGTCT-3′
TGF-βRII	Forward: 5′-GTCTACTCCATGGCTCTGGT-3′ Reverse: 5′-ATCTGGATGCCCTGGTGGTT-3′
VEGF	Forward: 5′-AGACCCTGGTGGACATCTTC-3′ Reverse: 5′-TGCATTCACATTTGTTGTGC-3′
GAPDH	Forward: 5′-TGAACGGGAAGCTCACTGG-3′ Reverse: 5′-TCCACCACCCTGTTGCTGTA-3′

### In vitro wound healing model (scratch assay)

A scratch assay was performed as previously described [35, 36]. Briefly, hGFs were seeded in 60-mm dishes in DMEM containing 10% FBS. On the following day, the cells were washed, and the medium was replaced with serum-free-DMEM for 24 h. Next, a straight scratch line was created across the cell monolayer using a sterile 200-µL pipette tip. The medium was then removed, and the cells were incubated in a medium containing DMSO or 1 µM curcumin for 24 h. Total RNA extraction was performed, and gene expression was evaluated by qPCR. In the ERK signaling inhibitor experiments, the cells were incubated with DMSO or 100 nM PD98059 (Cell Signaling Technology, Danvers, USA) for 1 h followed by DMSO or 1 µM curcumin treatment for 24 h.

### Statistical analysis

The data are presented as mean ± standard deviation of three independent experiments with gingival fibroblasts from three different subjects in each experimental condition (n = 9). Normality test was performed using the Shapiro–Wilk test. Normally distributed data were analyzed using the independent *t*-test or one-way ANOVA followed by Tukey’s Honestly Significant Difference test. *p* < 0.05 denoted statistical significance.

## Results

### Effects of curcumin on hGF viability

**Fig. 1:**
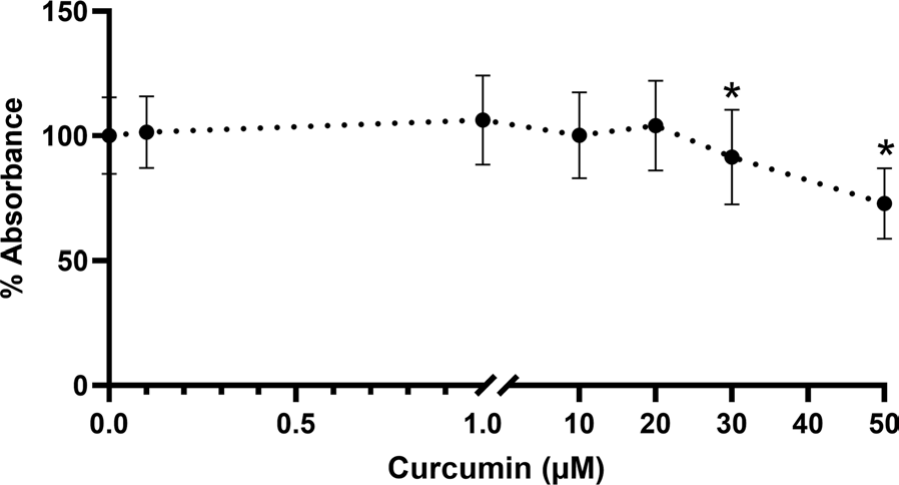
Cytotoxicity of curcumin in human gingival fibroblasts. Cells were seeded at 5 × 10^3^ cells per well in 96-well plates and treated with varying concentrations of curcumin or DMSO for 24 h. Cell viability was measured using the MTT assay. The data are presented as the mean ± standard deviation. *p < 0.05 compared to the control group

The cell viability of curcumin-treated HGFs was evaluated using the MTT assay. Figure 1 demonstrates that 0.1–20 µM curcumin did not alter cell viability, whereas 30 and 50 µM curcumin significantly induced concentration-dependent cytotoxicity (*p* < 0.05). Therefore, curcumin was used at concentrations of 0.1–20 µM in the subsequent experiments.

### Effects of curcumin on wound healing-related gene expression

**Fig. 2:**
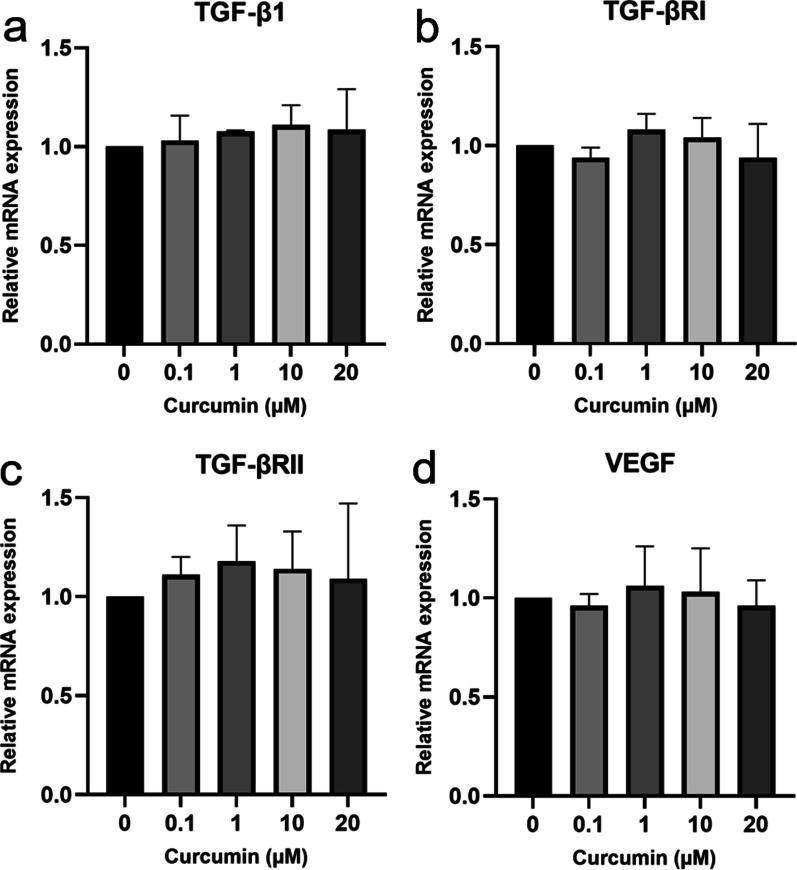
The expression of transforming growth factor (TGF)-β1 (**a**), type I TGF-β receptor (TGF-βRI) (**b**), type II TGF-β receptor (TGF-βRII) (**c**), and vascular endothelial growth factor (VEGF) (**d**) in human gingival fibroblasts in response to curcumin. Cells were seeded at 6 × 10^5^ cells per plate and treated with varying concentrations of curcumin or DMSO for 24 h. Gene expression was determined by quantitative polymerase chain reaction. The data are presented as the mean ± standard deviation

In unwounded gingival fibroblasts, 0.1–20 µM curcumin had no significant effect on TGF-β1, TGF-βRI, TGF-βRII, or VEGF expression (Fig. 2). However, our previous study identified 1 µM as the optimal curcumin concentration for inducing the expression of other genes, such as keratinocyte growth factor-1 and epidermal growth factor receptor. Therefore, 1 µM curcumin was used in our subsequent experiments [35].

### Curcumin induced TGF-β1, TGF-β receptors, and VEGF expression in the in vitro wound healing model

**Fig. 3:**
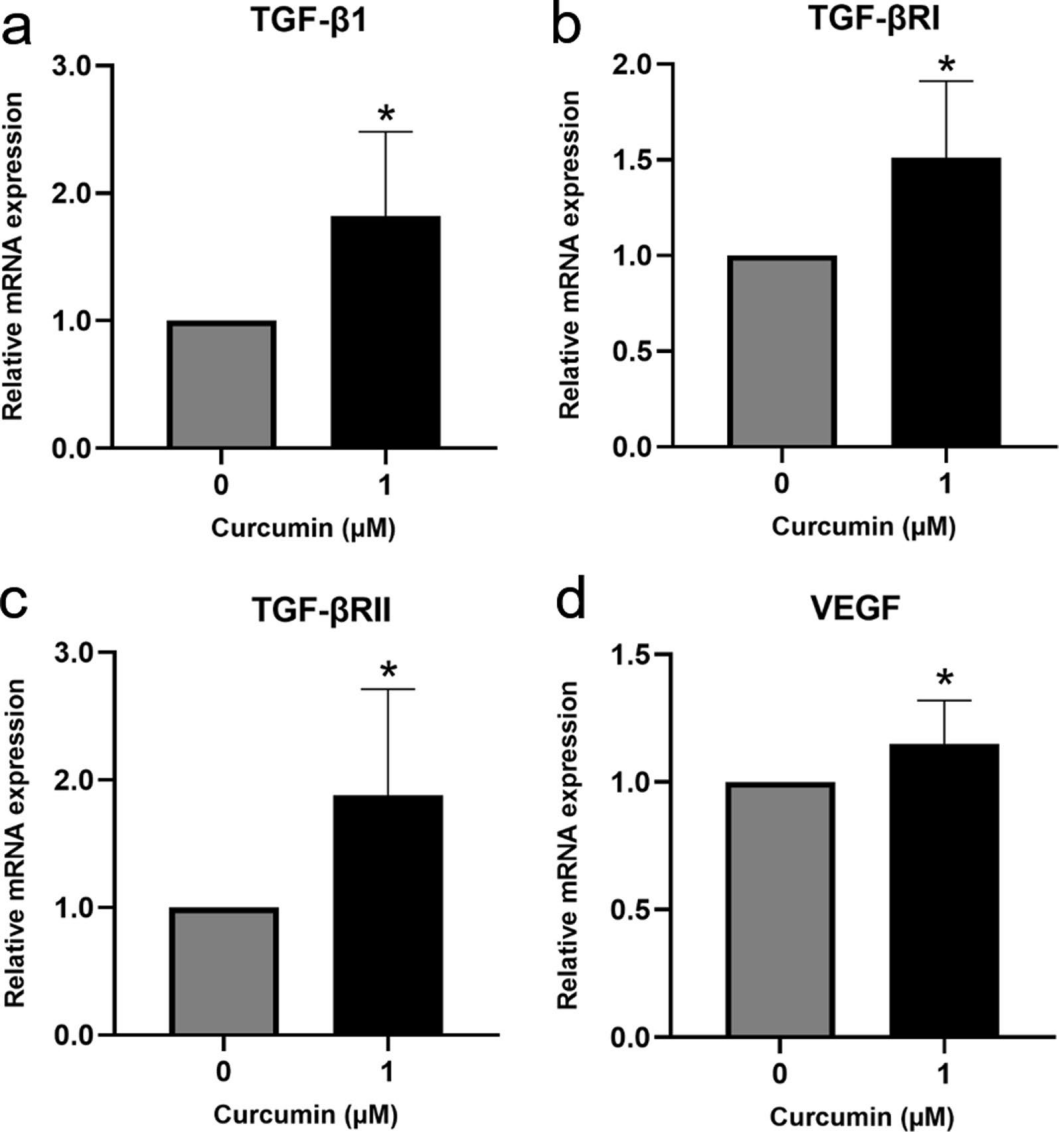
Curcumin induced transforming growth factor (TGF)-β1 (**a**), type I TGF-β receptor (TGF-βRI) (**b**), type II TGF-β receptor (TGF-βRII) (**c**), and vascular endothelial growth factor (VEGF) (**d**) expression in human gingival fibroblasts in the in vitro wound healing model. Cells were seeded at 6 × 10^5^ cells per plate, scratched after monolayer formation, and treated with 1 µM curcumin for 24 h. Gene expression was determined by quantitative polymerase chain reaction. The data are presented as the mean ± standard deviation. *p < 0.05 compared to the control group

To investigate the effect of curcumin in the in vitro wound healing model, a scratch assay was performed using hGF monolayers followed by treatment with 1 µM curcumin for 24 h. Curcumin significantly upregulated TGF-β1, TGF-βRI, TGF-βRII, and VEGF mRNA expression (*p* < 0.05) in the in vitro wound healing model compared to the findings in the vehicle-treated control (Fig. 3).

### ERK signaling inhibition decreased curcumin-stimulated gene expression in the in vitro wound healing model

**Fig. 4:**
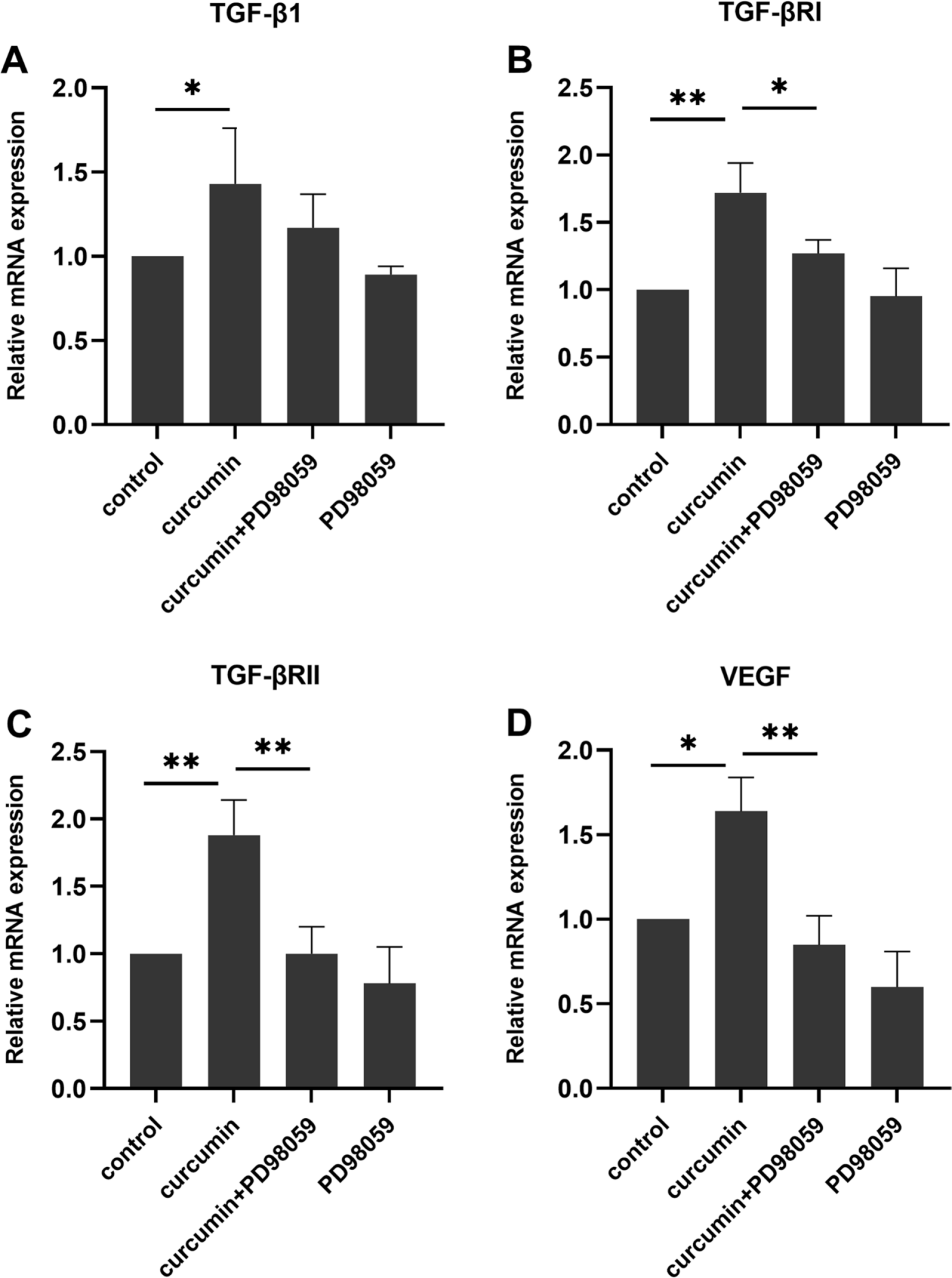
Effect of PD98059 on curcumin-induced transforming growth factor (TGF)-β1 (**a**), type I TGF-β receptor (TGF-βRI) (**b**), type II TGF-β receptor (TGF-βRII) (**c**), and vascular endothelial growth factor (VEGF) (**d**) expression in human gingival fibroblasts (hGF) in the in vitro wound healing model. Cells were plated at 6 × 10^5^ cells per plate in tissue culture dishes. The wounded hGF culture was treated with 100 nM PD98059 for 1 h prior to 1 µM curcumin treatment for 24 h. Gene expression was determined by quantitative polymerase chain reaction. The data are presented as the mean ± standard deviation. *p < 0.05. **p < 0.001

To determine whether ERK signaling is involved in curcumin-stimulated gene expression, the wounded hGF monolayer was incubated with PD98059 prior to curcumin treatment. PD98059 had no effect on curcumin-induced TGF-β1 expression (Fig. 4a). However, PD98059 significantly decreased curcumin-induced TGF-βRI, TGF-βRII, and VEGF mRNA expression (all *p* < 0.05, Fig. 4b–d).

## Discussion

In the present study, we first examined the cytotoxicity of curcumin on hGFs. The results indicated that 0.1–20 µM curcumin did not significantly affect hGF viability, whereas 30–50 µM curcumin significantly induced concentration-dependent cytotoxicity. Then, we demonstrated that curcumin did not significantly affect TGF-β1, TGF-βRI, TGF-βRII, or VEGF mRNA expression in unwounded hGFs. Interestingly, in the in vitro wound healing model, curcumin significantly stimulated TGF-β1, TGF-βRI, and TGF-βRII expression in hGFs.

Previous studies reported that TGF-β1 expression was remarkably lower in non-healing wounds than in normal healing ulcers [37, 38]. Moreover, many studies reported increased wound healing when the wound was treated with exogenous TGF-β1 [39–41]. The TGF-β receptors are localized on the cell surface of several cell types, including fibroblasts and endothelial cells [42, 43]. In previous research, TGF-β receptor expression increased after injury, with the highest expression observed on day 14 and declining by day 56 after wounding [44]. A previous study demonstrated that curcumin differentially regulated TGF-β and its receptors. Specifically, curcumin induced TGF-β1 and TGF-βRII expression in unimpaired and impaired healing cutaneous wounds, whereas TGF-βRI expression was increased only in impaired healing wounds 7 days after wounding [8]. We also reported the similar differential regulation of TGF-β and its receptors by curcumin in which curcumin increased the expression of TGF-β and its receptors only in the in vitro wound healing model but not in the unwounded gingival fibroblasts, supporting the concept that curcumin promotes healing in wounded tissues [8].

VEGF is a strong positive regulator of angiogenesis that stimulates endothelial cells for new blood vessel formation [45]. The VEGF receptor is expressed mainly on endothelial cells [46]. VEGF-A deficient mice exhibit delayed wound closure because of reduced vessel density [47]. In another study, curcumin enhanced blood vessel formation and promoted wound healing by increasing VEGF and TGF-β1 expression in diabetic rat granulation tissue [48]. In addition to cutaneous wounds, curcumin promoted indomethacin-induced gastric ulcer healing by increasing matrix metalloproteinase-2, TGF-β, and VEGF expression [49]. Correspondingly, we demonstrated for the first time that curcumin significantly increased VEGF expression in the in vitro hGF wound healing model. However, VEGF expression was not affected in the unwounded cell monolayer, suggesting that curcumin regulates gene expression in a controlled “as needed” manner because VEGF upregulation is unnecessary in unwounded tissues. However, the mechanism by which curcumin differentially regulates gene expression under physiological and wounded conditions remains to be elucidated.

Curcumin regulates gene expression in part by modulating the phosphorylation activity of MAPK [33]. The MAPK pathway has an important function in transducing extracellular signals to cellular responses [50]. The MAPK family comprises MAPK (also known as ERK), C-Jun N-terminal kinase/stress activated protein kinase, and p38 MAPK [50]. MAPK is involved in many cell functions, especially in regulating cell migration and proliferation [51, 52]. A previous study demonstrated that ERK signaling is important for the proliferative phase of cutaneous wound healing by promoting keratinocyte proliferation and migration [53]. Inhibition of either the ERK1/2 or p38 pathway resulted in delayed corneal epithelial wound healing [54]. In the present study, curcumin-stimulated TGF-βRI, TGF-βRII, and VEGF expression was significantly attenuated by an ERK signaling inhibitor, PD98059, while TGF-β1 expression was not affected. These data suggest that curcumin regulates TGF-βRI, TGF-βRII, and VEGF expression in hGFs by modulating the ERK pathway. Our findings are consistent with our previous report that curcumin-induced type I collagen and EGFR expression in hGFs also requires ERK activation [35]. However, most previous studies investigated the effect of curcumin in various cancer cell lines and observed that curcumin inhibited ERK signaling [33]. Taken together, these findings suggest that curcumin might differentially regulate ERK in normal cells and cancer cells; however, the exact mechanism requires further investigation.

Although the scratch assay used in this study is not an ideal wound healing model, it has been widely used to study the effects of many drugs and biological factors on keratinocyte and fibroblast migration [36, 55]. At the cellular level, scratching induces an increase in reactive oxygen species, Nrf2 protein, and stress response genes, including heat shock protein 70 and heme oxygenase-1, in breast cancer cells [36]. The antioxidative property of curcumin may reduce ROS production in the injured cells, which may be one of the mechanisms by which curcumin promotes wound healing. Further investigations are required to explore this possibility in hGFs.

Mechanical injuries from scratching cause the injured cells to produce chemical stimuli that diffuse to neighboring cells. In addition, scratching induces an increase in intracellular calcium in the injured cells which is transmitted through cell–cell junctions to nearby cells that are not damaged. This calcium wave propagates into cells that are distant from the wound edge [56]. Increased intracellular calcium activates multiple signaling pathways, resulting in alteration of gene expression [57]. These data suggest that mechanical injuries may alter gene expression not only in cells around the wound edge, but also in those located a long distance away. A previous study demonstrated that curcumin dose-dependently decreased the intracellular calcium level in colorectal carcinoma cells [58]. Whether curcumin could regulate the changes in intracellular calcium level induced by mechanical injuries requires further investigation. Because wound healing is a complex process and requires interaction between several cell types, the scratch assay using one cell type only mimics the mechanical injury to these cells. Owing to this limitation, further investigation is required to support our findings on the effect of curcumin on gene expression in this wound healing model.

## Conclusions

Curcumin significantly upregulated TGF-β1, TGF-βRI, TGF-βRII, and VEGF mRNA expression in the in vitro hGF wound healing model. The ERK pathway is crucial for curcumin-stimulated TGF-βRI, TGF-βRII, and VEGF mRNA expression. Further investigations are required to examine other mechanisms of curcumin that are responsible for promoting gingival wound healing. Our findings support the therapeutic potential of curcumin as a wound healing agent for treating gingival ulcers.

## Data Availability

The datasets generated and/or analyzed during the current study are available in the Figshare repository, https://doi.org/10.6084/m9.figshare.15073029.

## Abbreviations

hGFs: Human gingival fibroblasts
TGF-β1: Transforming growth factor beta 1
TGF-βRI: Type I TGF-β receptor
TGF-βRII: Type II TGF-β receptor
VEGF: Vascular endothelial growth factor
MTT: (3-(4,5-Dimethylthiazol-2-yl)-2,5-diphenyltetrazolium bromide)
ERK: Extracellular signal regulated kinase
MAPK: Mitogen-activated protein kinase
DMEM: Dulbecco’s Modified Eagle’s medium
FBS: Fetal bovine serum
PBS: Phosphate-buffered saline
DMSO: Dimethyl sulfoxide
GAPDH: Glyceraldehyde-3-phosphate dehydrogenase
